# Structural and functional studies of *Escherichia coli* aggregative adherence fimbriae (AAF/V) reveal a deficiency in extracellular matrix binding

**DOI:** 10.1016/j.bbapap.2016.11.017

**Published:** 2017-03

**Authors:** Rie Jønsson, Bing Liu, Carsten Struve, Yi Yang, René Jørgensen, Yingqi Xu, Håvard Jenssen, Karen A Krogfelt, Steve Matthews

**Affiliations:** a Institute for Science and Environment, Roskilde University, Roskilde, Denmark; b Department of Microbiology and Infection Control, Statens Serum Institut, Copenhagen, Denmark; c Centre for Structural Biology, Department of Life Sciences, Imperial College London, South Kensington, London, United Kingdom

**Keywords:** EAEC, Enteroaggregative *Escherichia coli*, AAF, aggregative adherence fimbriae, NMR, nuclear magnetic resonance, ECM, extracellular matrix, SPR, surface plasmon resonance, SDS-PAGE, sodium dodecyl sulphate polyacrylamide gel electrophoresis, Ig, immunoglobulin, CU, chaperone-usher, RMSD, root mean square deviation, ELISA, enzyme-linked immunosorbent assay, TOCSY, total correlation (TOCSY) spectroscopy, NOE, nuclear Overhauser effect, DMEM, Dulbecco's modified Eagle's medium, Aggregative adherence fimbriae, Agg5A, Chaperone-usher, Pilin, Donor strand complementation, *E. coli*, Fibronectin

## Abstract

Enteroaggregative *Escherichia coli* (EAEC) is an emerging cause of acute and persistent diarrhea worldwide. The pathogenesis of different EAEC stains is complicated, however, the early essential step begins with attachment of EAEC to intestinal mucosa via aggregative adherence fimbriae (AAFs). Currently, five different variants have been identified, which all share a degree of similarity in the gene organization of their operons and sequences. Here, we report the solution structure of Agg5A from the AAF/V variant. While preserving the major structural features shared by all AAF members, only Agg5A possesses an inserted helix at the beginning of the donor strand, which together with altered surface electrostatics, renders the protein unable to interact with fibronectin. Hence, here we characterize the first AAF variant with a binding mode that varies from previously described AAFs

## Highlights

• AAF/V expressing strain show no specificity to extracellular matrix (ECM) proteins compared to the uncoated wells.
• Agg5A share structural similarity to the two previously described AAF subunits AggA and AafA.
• Fibronectin binding is restored by substitution of a single aspartic acid to lysine.

## 1 Introduction

Enteroaggregative *Escherichia coli* (EAEC) is a subgroup of diarrheagenic *E. coli*, which is recognized as a major cause of diarrhea worldwide. EAEC is associated with acute diarrhea in children and adults living in developing and developed countries [1], [2], persistent diarrhea in children of developing countries [3] and in human immunodeficiency virus-infected persons [4], traveler's diarrhea [5] and outbreaks of diarrhea associated with ingestion of contaminated food/water [6], [7]. Furthermore, recent studies have implicated EAEC as the cause of urinary tract infections [8].

The EAEC strains are very heterogeneous and their pathogenesis is complex [9], [10]. Numerous putative virulence factors have been identified, but the clinical impact of these factors remain unclear. However, initial attachment to the intestinal mucosa is an essential step in the colonization and production of disease by EAEC [11]. The adherence of EAEC to the human intestinal mucosa requires expression of aggregative adherence fimbriae (AAFs), where adherence is characterized as a biofilm composed by aggregates of bacteria in association with a thick mucus layer [12], [13].

**Fig. 1:**
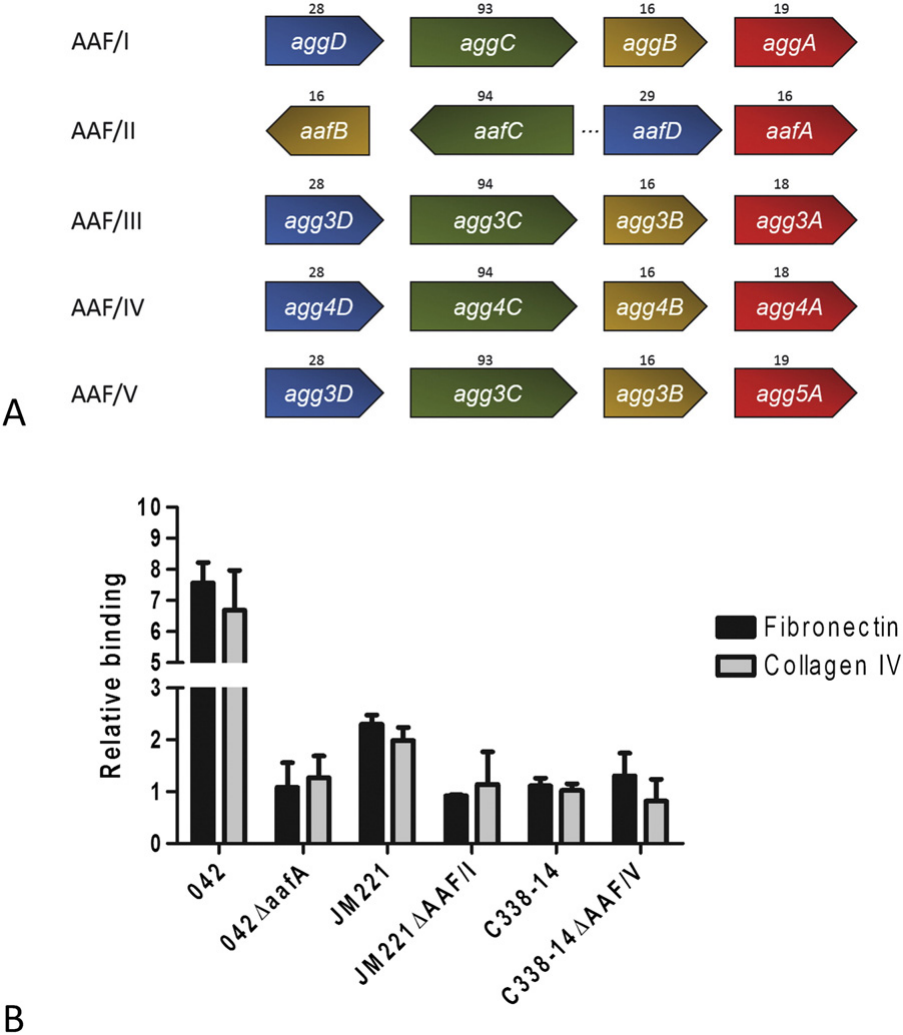
AAF/V shows no specificity towards fibronectin and collagen IV. (A) Organization of the operon encoding the AAF genes. The genes encoding the periplasmic chaperones are shown in blue, the outer membrane ushers in green, the minor pilin subunits in yellow and major pilin subunits in red. The numbers designate the molecular weight of encoded protein in kDa. (B) EAEC strain C338-14 harboring AAF/V does not bind to ECM molecules fibronectin and collagen IV, whereas the AAF/I and AAF/II expressing strains JM221 and 042 binds more to the ECM coated surfaces. The figure represents the relative fold binding with respect to the uncoated wells, where 1 equals no difference between adherence to the uncoated and the coated wells. The adherence of each strain was calculated as numbers of adhering bacteria relative to the total numbers of bacteria present in each well. The results are presented as the means ± standard errors of the means for at least triplicate samples and represent one of three independent experiments performed with similar results.

There are five known AAF variants (AAF/I-AAF/V) [14], [15], [16], [17], [18]. The AAF adhesins share a high degree of similarity in the organization of their operons, as well in the protein sequences of the chaperone-usher biogenesis machinery components (Fig. 1A). A greater degree of sequence divergence is exhibited in the genes that encode the major structural subunits [19]. Recently, the structural architecture of AggA (AAF/I) and AafA (AAF/II) were determined by X-ray crystallography and nuclear magnetic resonance (NMR) spectroscopy while AAF/IV minor subunit structure HdaB as determined by X-ray crystallography [20], [21], [22], [23]. In this work, it was shown that the major subunits of the AAFs assemble into linear polymers by donor strand complementation and the minor subunit forms the tip of the fimbriae, by accepting the donor strand from the terminal major pilin subunit [22].

Studies have previously shown that the archetype EAEC strain 042 expressing AAF/II binds to several major extra cellular matrix (ECM) proteins present in the intestinal epithelium, such as fibronectin, laminin, and type IV collagen [24]. From these data as well as validation by NMR and surface plasmon resonance (SPR), it was suggested that the AAFs have evolved an electrostatic mechanism for binding to host cell receptors using a patch of positively charged residues [22].

Though several studies support a role for AAF in EAEC pathogenesis, the cellular receptors for these fimbrial structures are still unknown. In this study, we report NMR studies of the monomeric, donor-strand complemented major pilin subunit of Agg5A, the newest member of the AAF family, which was recently shown to be very prevalent among EAEC strains isolated from Danish travelers with diarrhea (12%) and from children in Mali with diarrhea (13%) [18]. From the structure and results of binding studies, we show that Agg5A possess unique properties compared to the two AAFs previously described. Whereas AafA and AggA interact with fibronectin due to electrostatic interactions, Agg5A has evolved to include an insertion upstream of the donor strand which would represent the linker between polymerized subunits in the fibril. This feature together with altered electrostatic characteristics abolish binding to fibronectin as well as other ECM molecules. Based on the structure of Agg5A, we performed mutagenesis to successfully introduce fibronectin binding back into Agg5A. Our results show that Agg5A displays significantly differences from AafA and AggA, suggesting an evolutionary adaption to an alternative host receptor.

## 2 Results and discussion

### 2.1 Receptor binding of AAF/V

The major pilin subunits of AAF/I and AAF/II (AafA and AggA) have previously been shown to share the common receptor fibronectin. To examine if fibronectin is also a receptor for AAF/V, we tested the two wildtype reference strains 042 and C338-14 encoding AAF/II and AAF/V and their respective AAF deletion mutants. Moreover, we also included the AAF/I reference strain JM221 and its respective AAF/I mutant, which has previously been shown to produce biofilm and adherence to intestinal cells [8], [25].

The EAEC strains were added to 24-well plates either coated with the purified ECM proteins fibronectin, collagen IV or the uncoated control. Whereas JM221 (AAF/I) and 042 (AAF/II) exhibited high binding to the purified ECM proteins fibronectin and collagen IV and significant less binding to the uncoated surface, C338-14 expressing AAF/V showed no specificity to either of the ECM proteins compared to the uncoated wells (Fig. 1B). The AAF mutant strains failed to adhere to all surfaces. We repeated the pull-down method used previously [24], to confirm the findings described above. Cultures of bacteria were incubated with fibronectin for 3 h followed by extensively washing. Lysis of the bacterial cells in the presence fibronectin were analyzed by SDS-page and confirmed that fibronectin did not bind to the AAF/V expressing strain, while it did to the wildtype expressing AAF/II (data not shown).

### 2.2 The solution structure of self-complemented Agg5A

**Fig. 2:**
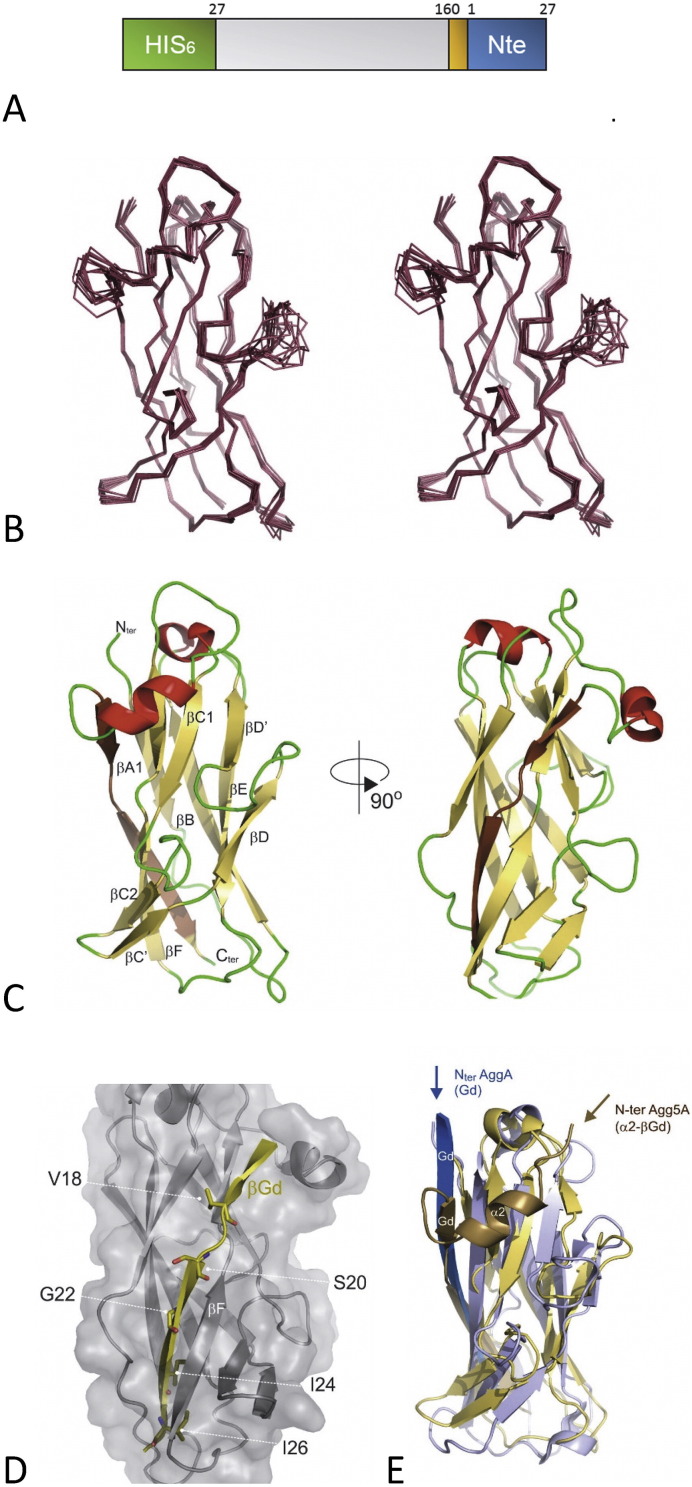
Solution structure of Agg5AdscA. (A) Schematic representation of the Agg5A donor strand construct. The positions of residues flanking the mature sequences are numbered. N-terminal His-tag is colored in green, DNKQ linker in orange and the N-terminal extension (Nte) in blue. (B) Final ensemble of 10 NMR structures shown in stereo. (C) Cartoon representation of Agg5AdscA with β-strands, α-helices and loops colored in yellow, red and green respectively. The C-terminal self-complementing donor strand is shaded in darker yellow color. N- and C-termini are labelled according to the dsc construct. (D) Transparent surface representation of Agg5AdscA with the donor strand shown in yellow together with key interacting side-chains in sticks. (E) Cartoon representation of the best fit superposition of Agg5AdscA with the crystal structure of AggAdscA. For Agg5A the backbone cartoon is colored in yellow and AggA is in blue, while the self-complementing donor strands are shaded in darker color. The orientation and direction the N-termini (i.e. the self-complementing donor strands, Gd) are shown as they would be arranged in native polymerized subunits.

We next we determined the high resolution structure of a self-donor strand complement form of the pilin protein Agg5A. The N-terminal donor strand was removed and appended to the C-terminus of the Agg5A major subunit with an intervening ‘DNKY’ turn in an analogous fashion as that used for structural studies of AggA and AafA [22] (Fig. 2A). Agg5AdscA purified in soluble form, suggesting that the donor strand sequences were correctly located in the subunit to produce a stabilized, monomeric form. Crystallization of Agg5AdscA was screened by the sitting-drop vapour diffusion method, but failed to identify any promising conditions. The NMR spectra of Agg5A exhibited excellent dispersion and line-widths which confirm the monomeric status of Agg5A. Therefore, solution structure determination was carried out using multidimensional NMR spectroscopy (Fig. 2B; Table S1; protein Data Bank [PD] ID code 5LVY and Biological Magnetic Resonance Bank [BMRB] ID code 34042). The overall architecture of Agg5A is a classical Ig-like fold that consists of two β-sheets packed against each other in a β-sandwich (Fig. 2C).

**Fig. 3:**
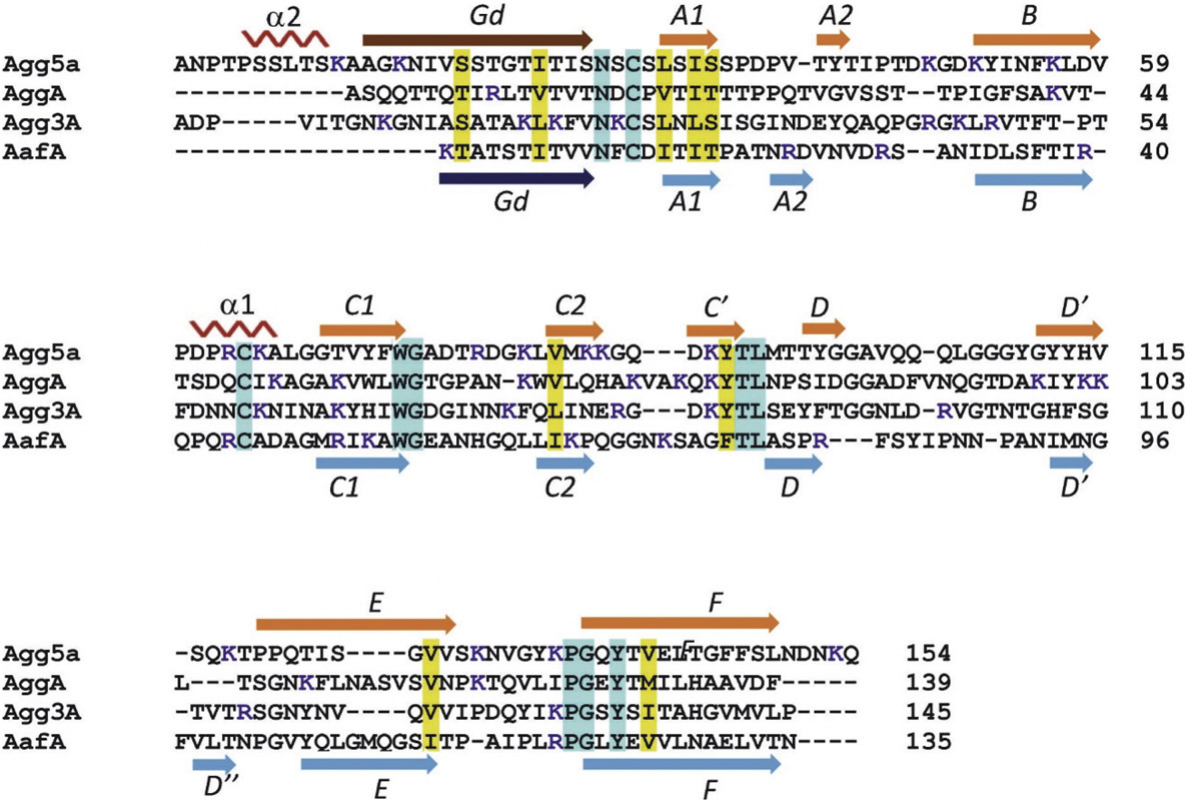
Sequence alignment of Agg5A with homologues to the other AAF family of chaperone-usher variants. The location of the helices is shown as squiggles and beta strands are shown as arrows. The secondary structure is based on the solution structure of Agg5Adsc determined by NMR and the crystal structure of AggAdscA. Residues numbers are shown for the end residues of the sequence lines. Cyan shading represents identical residues and yellow indicated conservation in the amino acids of the similar nature across the four sequences. Positively charged residues are shown in a blue font. Blue arrows are the beta strands in AafA while orange arrows are strands in Agg5A.

The donor strand (Gd) interactions with its neighboring F strand in the pilus subunit are shared with other chaperone-usher (CU) systems such as for AggA, AafA [22], Saf [26] and the F1 antigen [27], which are all members of the FGL family, in which the chaperone component comprised long F and G strands and they assemble linear polymers of just one or two subunit types. The Gd strand forms that edge of the CDF β-sheet, whereas in the P-pilus and type 1 fimbriae it interacts intimately with A and F strands (Fig. 2D)[28]. As reported earlier this feature distinguishes the FGL from FGS CU systems as a longer chaperone G′ strand is required to stabilize the entire length of the subunits F strand [29]. The overall similarity between Agg5A and either AggA or AafA, which are the closest structural homologues, displays an RMSDs of 2.7 Å over 123 C_α_ atoms and 3.5 Å over 120 C_α_ atoms, respectively (Fig. 2E). Significant conformational differences occur in the loops decorating the CDF β-sheet face; particularly in the loop regions C2-C′ and D-D′, together with the beginning of N-terminal donor strand Gd (Fig. 2E). The rest of the structural features are largely conserved, including the disulfide bond between C33–C64 connecting the α1 helix to the start of the subunit fold (Fig. 3).

Two conserved surface exposed residues were noted in the previously determined structures, suggesting a role in the adherence of the AAF/Afa family (Fig. 3). These were Trp59 at the end of strand C1 and neighboring basic residue at 55 within C1 (numbering according AggA in Fig. 3). Trp59 was shown to not be involved in fibronectin recognition, but the basic position at 55 along with a positive surface patch encompassing three closely spaced Lysine residues (Lys73, Lys76 and Lys78) in the C2-C′ loop play an active role in fibronectin binding. In Agg5A only the tryptophan is absolutely conserved in sequence position. Our structure also reveals that a new helical feature is formed between Pro5 and Ser10 that lies across this region. This helix, termed α2, is located at the N-terminus of Agg5A, joins the donor strand thereby extending this region by an additional 12 residues (Fig. 2, Fig. 3). The Agg5A helical insertion and its packing along the CDF β-sheet face shifts the location of its N-terminus to above the C1 strand. This feature has not been seen on other chaperone-usher pilins and it is therefore conceivable that this arrangement could alter the relative subunit packing within fimbriae or occlude the binding capability of the subunit. Election microscopy of AAF fimbriae showed an extended arrangement of subunits, which was envisaged to provide a continuous band of positive charge running along the fiber and mirrored the extended nature of fibronectin [22]. This helical insertion would introduce steric clashes with such an extended fibronectin molecule thereby reducing it affinity.

### 2.3 Fibronectin as a receptor for AAFs

**Fig. 4:**
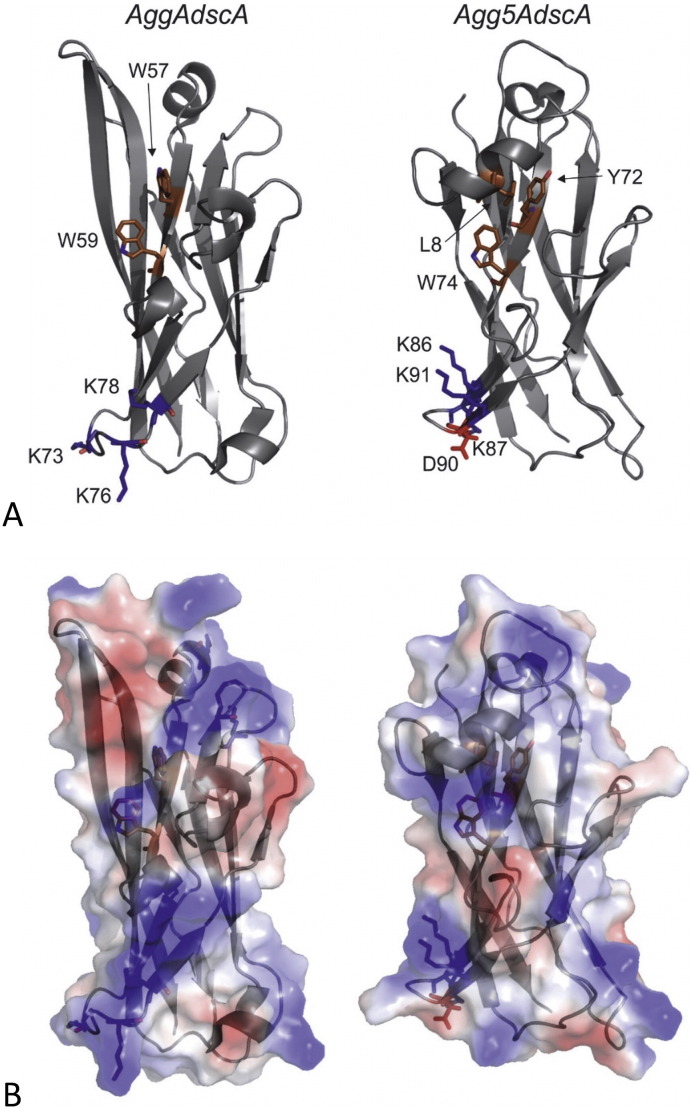
Surface properties of Agg5A and AggA. (A) Left – cartoon representation of AggA (PDB ID: 2MPV) electrostatic surface, calculated using the Pymol plugin [36], showing basic residues that play a role in fibronectin binding and the surface exposed tryptophans. Right – cartoon representation of Agg5A showing the side chains with the equivalent regions. (B) Left – surface representation of AggA showing its surface electrostatic properties. Right – surface representation of Agg5A showing its surface electrostatic properties.

**Fig. 5:**
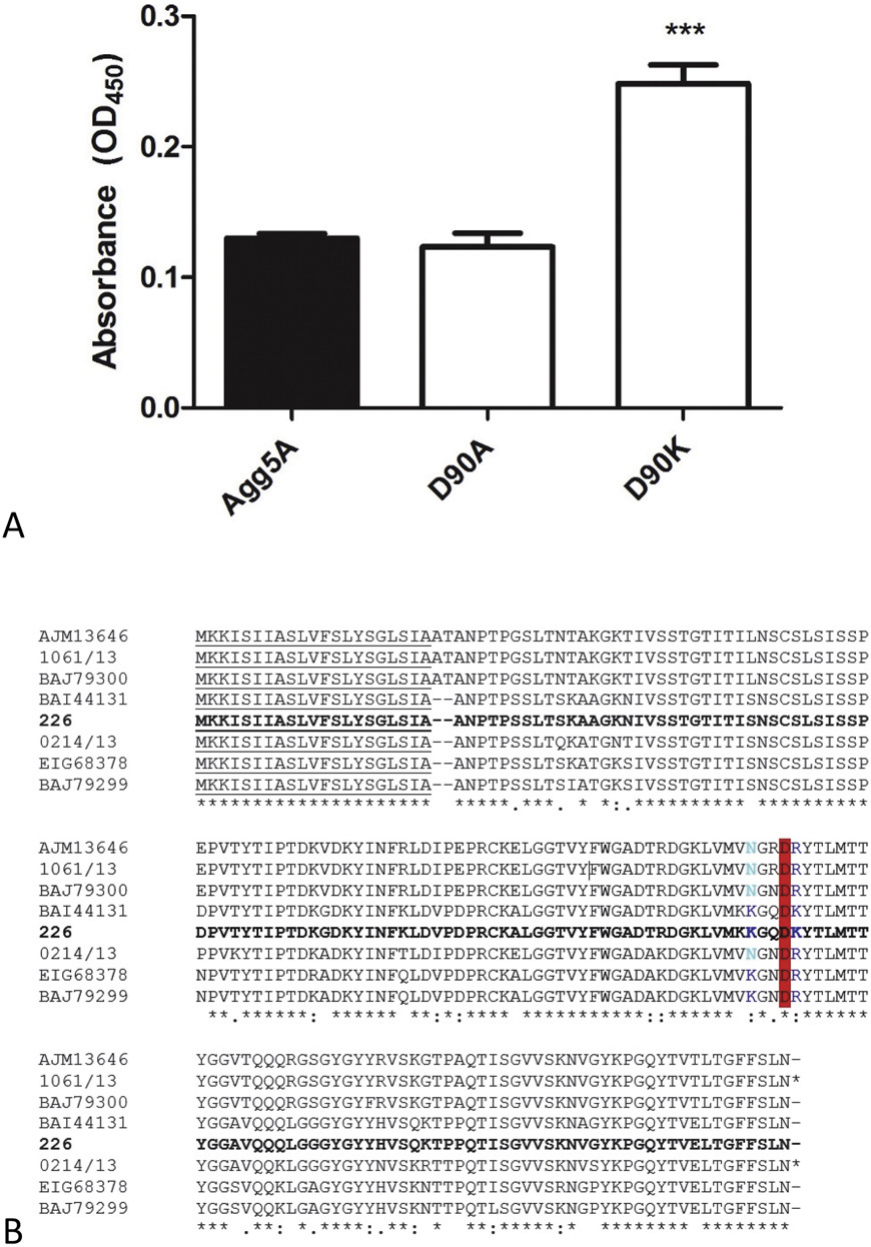
Fibronectin binding is abolished by aspartic acid. (A) Change of aspartic acid to lysine restores fibronectin binding of Agg5A (*P* < 0.001). Proteins were added at 10 μg/ml to wells of 96-wells plate coated with 25 μg/ml fibronectin, and the binding was determined by ELISA using anti-Agg5A antiserum. The bars represent the means of three experiments ± standard errors. (B) Conservation is observed in the aspartic acid among 8 Agg5A variants. The Asp90 is conserved among Agg5A variants. Sequence alignment of the amino acids of 8 Agg5A variants. The conserved aspartate at position 90 is highlighted in red, whereas the positive amino acids responsible for fibronectin binding are blue. Asterisks and points represent identical and similar residues, whereas gaps (-) have been inserted to optimize the alignment.

It has been established that fibronectin is a common receptor for all AAF variants tested to date, and single subunits of AggA and AafA are able to bind with low micromolar disassociation constants [22], [24]. Polymers of AAF subunits would mediate a tight bacterial association with the ECM proteins by establishing multipoint interactions with fibronectin. It was also established that binding by AAF/I and AAF/II is driven by electrostatic interactions, as the mutagenesis of proximal pairs of lysines reduced their binding affinity significantly [22]. Most strikingly, a triple mutant of the positive patch of AggA delineated by three closely positioned lysines in the C2-C′ loop (Lys73, Lys76, and Lys78) abrogates fibronectin binding completely. Despite the lack of a fibronectin interaction with AAF/V fimbriae, and although these lysines are not totally conserved, some similar electrostatic characteristics are present in Agg5A, for example the Lys73, Lys76, and Lys78 residues in the C2-C′ loop of AggA is represented by Lys86, Lys87 and Lys91 (Fig. 4A). In contrast though, a negatively charged aspartate is also present at position 90 in Agg5A, where this region is exclusively basic in AggA. Asp90 also lies with the C2-C′ loop and in our structure would be exposed on the fibronectin binding surface of AggA (Fig. 4A & B). We reasoned that if we remove the negative charge of this residue fibronectin binding would be restored. To test this hypothesis, we performed site directed mutagenesis on Asp90 to alanine and lysine. The wildtype and two mutants were tested with a fibronectin ELISA using rabbit antiserum raised against Agg5A. Mutation of Asp90 to Alanine has no effect on fibronectin binding whereas swapping the charge to a lysine partially restored an interaction confirming that this residue is important for binding to fibronectin (Fig. 5A). A collagen IV ELISA was also tested, however no significant binding was observed between the wildtype and mutants (data not shown).

### 2.4 Asp90 is conserved among Agg5A variants

We next aligned Agg5A sequences derived from different EAEC strains, since a previous study showed variation ranging from 83% to 100% among the isolates [18]. Interestingly, although amino acid variation was observed between the strains, the Asp90 residue is conserved among all the variants, further indicating that this residue indeed is important for the functionality of Agg5A and the absence of fibronectin recognition (Fig. 5B).

Taken together our results provides new insights into the adhesion and pathogenesis of the AAFs. Whereas binding to ECM proteins is observed for all other AAFs, our data shows that AAF/V does not bind to fibronectin and this is likely due in part to the introduction of the AAF/V-conserved negatively charged amino residue Asp90, which interrupts the continuous band of positive charge displayed on the βC-βD surface of AggA and alters its charge distribution (Fig. 4). The mutagenesis data and binding studies revealed that a mutation introduced at position Asp90 to a positively charged residue lysine is able to partially restore binding to fibronectin.

The high prevalence of Agg5A among clinical EAEC isolates indicates that the conserved mutation at Asp90 is important for changing the binding specificity of AAF/V compared to the other AAFs, and may promote a distinct pattern of host colonization. This could be via increased adhesion to alternative host receptors enabling the bacteria to colonize other host niches or by decreased recognition by the host immune system. Since Agg5A does not bind to the same substrates as the other AAF variants, further studies are needed to identify the receptor for Agg5A. Furthermore, the prevalence of Agg5A needs to be further investigated, since many studies are still only examining for AAF/I-AAF/IV [30], [31].

## 3 Materials and methods

### 3.1 Bacterial strains, plasmids and growth conditions

Bacterial strains and plasmids used in this study is listed in Table 1. Prototype EAEC strains included in this study were AAF/I producing strain JM221, AAF/II producing strain 042 and AAF/V producing strain C338-14. Overnight cultures of bacteria grown in Luria-Bertani (LB) broth at 37 °C containing antibiotics where appropriate: 100 μg/ml ampicillin and 50 μg/ml kanamycin. Prior to binding studies, bacteria were subcultured in Dulbecco's Modified Eagle Medium (DMEM) with 0.45% glucose/l.

### 3.2 Protein preparation

The dsc-Agg5A was constructed using the translated nucleotide sequence of Agg5A (Accession number SRA055981) as previously described [20], [32]. The sequence encoding for the dsc-Agg5A was ordered from Genscript and ligated into the pQE-30 vector (Qiagen, Venlo, Netherlands) via *Bam*HI and *Hin*dIII restriction sites and expressed in *E. coli* strain M15 cells with pREP4 plasmids. The cells were grown in either LB or M9 minimal medium supplemented with ^15^NH_4_Cl and ^13^C-glucose (Cambridge Isotope Laboratories) and induced with 1 mM isopropyl β-d-1-thiogalactopyranoside (IPTG) when the OD_600_ reached 0.6, which was followed by overnight incubation at 37 °C before harvesting by centrifugation. The cells were lyzed by sonication under denaturing conditions before being purified with Ni-NTA (Qiagen). The eluate was first dialyzed against 50 mM sodium acetate pH 5, 50 mM NaCl, 1 M urea, which was followed by a second dialysis against the same buffer but without urea. Agg5a was further purified by gel filtration using a Superdex 75 gel-filtration column (GE Healthcare). Monomeric Agg5A fractions were pooled and concentrated to 0.5 mM for the NMR experiments.

### 3.3 NMR structure determination

Spectral assignments were completed using our in-house, semi-automated assignment algorithms and standard triple-resonance assignment methodology [33]. H_α_ and H_β_ assignments were obtained using HBHA (CBCACO)NH and the full side-chain assignments were extended using HCCH-total correlation (TOCSY) spectroscopy and (H)CC(CO)NH TOCSY. Three-dimensional ^1^H-^15^N/^13^C NOESY-HSQC (mixing time 100 ms at 800 MHz) experiments provided the distance restraints used in the final structure calculation. The ARIA protocol [34] was used for completion of the NOE assignment and structure calculation. The frequency window tolerance for assigning NOEs was ± 0.04 ppm and ± 0.06 ppm for direct and indirect proton dimensions and ± 0.6 ppm for both nitrogen and carbon dimensions. The ARIA parameters p, Tv and Nv were set to default values. 144 dihedral angle restraints derived from TALOS were also implemented [35]. The 10 lowest energy structures had no NOE violations > 0.5 Å and dihedral angle violations greater than 5^o^. Although structure calculations readily converged without the introduction of manual assignments, a systematic check of automatically assigned NOEs was carried out. The 10 structures were deposited to PDB (accession number: 5LVY) and statistics are shown in Table S1.

### 3.4 Bacterial binding to fibronectin and collagen IV

Quantification of bacterial binding to ECM (Sigma-Aldrich, St. Louis, MO) proteins was performed as previously described with modifications [24]. Briefly, wells of microtiter plates were coated with solution of 25 μg/ml of protein (fibronectin from human plasma or collagen IV from human placenta (Sigma)) in 100 mM Tris-HCl buffer, pH 8.0 overnight at 4 °C. Plates were washed 5 times with phosphate-buffered saline (PBS) to remove unbound protein and blocked with 5% milk in PBS for 4 h at 4 °C. 1 ml of Dulbecco's modified Eagle's medium (DMEM) with 0.5% glucose medium containing 1 × 10^8^ bacteria grown at 37 °C for 4 h were added to the wells. For quantification of the total number of bacteria, Triton X-100 (0.5% final concentration) was added to wells containing both well-associated and non-adhering bacteria. For quantification of adhering bacteria using other wells, non-adhering bacteria were removed by washing and the adhering bacteria were removed from the wells with 0.5% Triton X-100. Serial dilutions of bacteria were plated and colonies counted the following day. The figure represents the relative fold binding with respect to the uncoated wells, where 1 equals no difference between adherence to the uncoated and the coated wells. The adherence of each strain was calculated as numbers of adhering bacteria relative to the total numbers of bacteria present in each well.

### 3.5 Pull-down analysis

The strains were grown in DMEM/0.5% glucose and approximately 1 × 10^8^ bacteria were collected by centrifugation and washed twice in PBS. Nonspecific binding was blocked for 1 h at 37 °C in PBS containing 3% BSA. The cells were collected by centrifugation, resuspended in 100 μg/ml fibronectin and incubated for 3 h at 37 °C. Unbound fibronectin was removed by washing the cells 5 times in PBS. Cell-associated fibronectin was detected by separation of whole-cell lysates on 10% SDS-page, followed by staining with Coomassie blue (Thermo Scientific, Waltham, MA).

### 3.6 Site directed mutagenesis

Site directed mutagenesis was performed according to the QuickChange protocol (Stratagene, Cedar Creek, TX) with the *Pfu*Turbo high fidelity polymerase. 50 ng of pQE30-dscAgg5A plasmid was combined with 10 pmol of primers. The primers used for this purpose were 5′-GGTCATCAGGGTATATTTAGCCTGGCCTTTCTTCATGAC-3′ and 5′-GTCATGAAGAAAGGCCAGGCTAAATATACCCTGATGACC-3′ for the alanine substitution, and primers for substitution of lysine were 5′-GTGGTCATCAGGGTATATTTCTTCTGGCCTTTCTTCATGACCA-3′ and 5′-TGGTCATGAAGAAAGGCCAGAAGAAATATACCCTGATGACCAC-3′.

All constructs were verified by sanger sequencing at Macrogen (Seoul, Korea). The mutated plasmid was transformed into the M15 harboring the pREP4 plasmid and selectively grown on LB-agar with ampicillin and kanamycin.

### 3.7 Solid phase binding assay

Polysorp™ Microtiter plates (Thermo Scientific) were coated with a solution of 25 μg/ml of fibronectin/collagen IV in 100 mM Tris-HCl buffer, pH 8.0, overnight at 4 °C. Unbound protein was removed by washing the plates eight times with PBS containing 0.05% Tween and was subsequently blocked with 3% bovine serum albumin (BSA) in PBS for 1 h at room temperature. The blocking buffer was removed, and the wells washed five times prior to the addition of 100 μl protein (10 μg/ml) followed by incubation for 3 h at room temperature. Anti-Agg5A antiserum raised in rabbits (diluted 1:2000) was used to detect the bound protein and anti-rabbit horseradish peroxidase conjugate (1:1000) (Agilent Technologies, Santa Clara, CA) was added following another wash step with PBS + Tween. The peroxidase activity was detected with the addition of TMB plus solution (KPL, Gaithersburg, MD). Optical densities were read at 450 nm with a 96-well plate reader. To analyze the binding data, the background absorbance from wells only containing the protein buffer was subtracted from the absorbance in the test wells.

### 3.8 Statistical analysis

Statistical significance between means were analyzed using the unpaired Student's *t*-test with a threshold *P* value of 0.05 with GraphPad Prism v6.00 (GraphPad Software, San Diego, CA). Values are expressed as the means of three experiments with one standard deviation error.

The following is the supplementary data related to this article.

Table S1 NMR data statistics for Agg5A (pdb 5LVY). Table S1

## Transparency document

Transparency document. Image 1

## Figures and Tables

**Table 1:** Strains used in this study.

Strains	Description	Reference
042	Wildtype EAEC strain expressing AAF/II	[37]
042 ∆* aafA 3.4.14*	042 with a Tn*phoA* inserted into the aafA gene	[15]
JM221	Wildtype EAEC strain expressing AAF/I	[25]
JM221 ∆* AAF/I*	JM221 in which a kanamycin cassette was inserted into the AAF/I cluster	[11]
C338-14	Wildtype EAEC strain expressing AAF/V	[18]
C338-14 ∆* AAF/V*	C338-14 in which a kanamycin cassette was inserted into the AAF/V cluster	[18]
M15pREP4	*E. coli* expression strain harboring a pREP4 plasmid for regulating expression from pQE vectors	[38]
