# Supplementary material for: Structural and functional studies of Escherichia coli aggregative adherence fimbriae (AAF/V) reveal a deficiency in extracellular matrix binding
Source: Biochim Biophys Acta. 2017 Mar;1865(3):304–11. doi: 10.1016/j.bbapap.2016.11.017 (PMC5289312; doi:10.1016/j.bbapap.2016.11.017)
Supplement: Table S1 — NMR data statistics for Agg5A (pdb 5LVY). [file mmc1.pdf]

Table S1. NMR data statistics for Agg5A (pdb 5LVY)

**NMR Experimental restraints**	
NOE	
Total	2482
Intra-residue	811
Inter-residue	1671
Sequential ($\lvert i-j \rvert$=1)	497
Short range (2<$\lvert i-j \rvert$<3)	175
Medium range (4<$\lvert i-j \rvert$<5)	38
Long range ($\lvert i-j \rvert$>5)	961
Dihedral angle	
φ	110
ψ	110
**Structure calculation statistics**	
Violations (mean and SD)	
Distance constraints (Å)	0.016 ± 0.017
Dihedral angle constraints (°)	0.52 ± 0.051
Maximum dihedral angle violation (°)	0.51
Maximum distance constraint violation (Å)	0.23
Deviations from idealized geometry	
Bond length (Å)	0.0020 ± 0.0003
Bond angle (°)	0.373 ± 0.008
Impropers (°)	0.306 ± 0.017
Average Pairwise rmsd (Å)	
Heavy	0.53 ± 0.07
Backbone	0.22 ± 0.02
